# *Amomum cardamomum* L. ethyl acetate fraction protects against carbon tetrachloride-induced liver injury via an antioxidant mechanism in rats

**DOI:** 10.1186/s12906-016-1121-1

**Published:** 2016-05-31

**Authors:** Dong-Woo Lim, Hyuck Kim, Ju-Yeon Park, Jai-Eun Kim, Jin-Young Moon, Sun-Dong Park, Won-Hwan Park

**Affiliations:** Department of Pathology, College of Korean Medicine, Dongguk University, Dongguk-Ro 32, Goyang, 10326 Republic of Korea; Department of Diagnostics, College of Korean Medicine, Dongguk University, Dongguk-Ro 32, Goyang, 10326 Republic of Korea; Department of Acupuncture and Meridian, College of Korean Medicine, Dongguk University, Dongguk-Ro 32, Goyang, 10326 Republic of Korea; Department of Prescription, College of Korean Medicine, Dongguk University, Dongguk-Ro 32, Goyang, 10326 Republic of Korea

**Keywords:** *Amomum cardamomum*, Carbon tetrachloride, Oxidative stress, Hepatic injury, Antioxidant property

## Abstract

**Background:**

Medicinal herb-derived drug development has become important in the relief of liver pathology. *Amomun cardamomum* is traditionally used therapeutically in Korea to treat various human ailments including dyspepsia, hiccupping, and vomiting. We investigated to assess the protective effect of *A. cardamomum* on carbon tetrachloride (CCl_4_)-induced liver damage through antioxidant activity in hepatic tissues of Sprague–Dawley rats.

**Methods:**

Antioxidant properties of different fractions from *A. cardamomum* from ethanol extracts were evaluated by an *in vitro* free radical scavenging systems. The protective effect of the ethyl acetate fraction from *A. cardamomum* (EAAC) against CCl_4_-induced cytotoxicity was determined by a cell viability assay using HepG2 hepatocarcinoma cells. In vivo study, the influence of EAAC concentrations of 100 and 200 mg/kg following CCl_4_-induced hepatic injury was assessed. Serum levels of glutamic oxaloacetic transaminase (GOT), glutamic pyruvic transaminase (GPT), and alkaline phosphatase (ALP) were determined, as was lipid peroxidation (malondialdehyde, MDA). Effect of EAAC on liver detoxification enzymes including superoxide dismutase (SOD), total glutathione (GSH), and glutathione S-transferase (GST) activity was measured in rat liver homogenates. Liver cytochrome P450 (CYP2E1) expression level was determined by quantification of mRNA.

**Results:**

Phytochemical analysis of *A. cardamomum* indicated that EAAC was enriched in total polyphenol and total flavonoid. Most of the tannins were confined to the hexane fraction. Hepatoprotective properties of EAAC were evident, with significantly reduced serum levels of GOT, GPT, and ALP compared with the control group. Improved hepatic antioxidant status was evident by increased SOD, GSH, and GST enzymes in rat liver tissue. Liver lipid peroxidation induced by CCl_4_ was apparent by increased intracellular MDA level. EAAC suppressed lipid peroxidation as evidenced by the significant decrease in MDA production. Expression of CYP2E1 was also significantly decreased at the higher concentration of EAAC, indicating the hepatoprotective efficacy of EAAC on acute liver damage.

**Conclusion:**

These results indicated that EAAC has a significant hepatoprotective activity on CCl_4_-induced acute hepatic injury in rats, which might be derived from its antioxidant properties and CYP2E1 downregulation.

## Background

The liver is crucial in metabolizing xenobiotics through various mechanisms that involve numerous detoxification enzymes and antioxidant activity. Accordingly, the liver is continuously exposed to harmful oxidative stresses that impair cell function, which trigger several liver diseases [1, 2]. Liver diseases remained a major global health burdens and medical issue [3]. Oxidative stress is defined as an imbalance between the systemic manifestation of reactive oxygen species (ROS) and the antioxidant defenses [4]. Oxidative stress is crucial in the pathogenesis of liver diseases including fibrosis and liver cirrhosis [5–9]. ROS are chemically reactive molecules containing oxygen that normally function in cellular responses in signal transduction to sustain life and as part of host defenses against various infections [10]. However, excessive production of free radicals including superoxide, hydroxyl radical, lipid free radical, and nitric oxide leads to damage in certain diseases of the liver [11]. Inhibition of free radicals has been linked to the alleviation of liver disorders [12].

Carbon tetrachloride (CCl_4_) can induce free radical toxicity and has been used as a hepatotoxin in diverse liver disease models [13, 14]. CCl_4_ is converted into reactants through the formation of reactive intermediates including trichloromethyl radicals (CCl_3_·, CCl_3_OO·) and free radicals by cytochrome P450 (CYP 450) [15]. These free radicals and related oxidative stresses induce the deformation of cellular macromolecules, and increases lipid peroxidation, protein degeneration, and genomic mutations in human liver tissue [16]. A member of the CYP family, CYP2E1, is involved in these reactions and in the metabolism of xenobiotics like ethanol. CYP2E1 is regulated by endogenous factors and foreign compounds [17, 18]. Suppression of CYP2E1 has reduced liver damage in various experimental models including in vitro and in vivo systems [19–21].

The side effects of modern synthetic drugs used to treat liver diseases remains unclear. Traditional medicines and plant-derived drugs might be an attractive alternative in the prevention and treatment of hepatic disorders [12, 22]. *Amomum cardamomum* L., a member of Zingiberaceae family, can be distinguished from large cardamom native to southern India and is nowadays cultivated widely in tropical regions. The plant seeds are widely used as a spice in many countries and traditionally as a therapeutic for relief of dyspepsia, hiccupping, vomiting, and alcohol detoxification [23]. The previous studies have reported that seed of *A. cardamomum* and its active components had antioxidant and anti-inflammatory activities [24, 25]. Important essential oils constituents including terpenes have been reported in this plant [26]. Another reports suggest that essential oils treatment containing α, β-pinene, d-camphor, and 1,8-cineole inhibited liver injury in animal models [27–29]. 1,8-Cineole, the bicyclic monoterpene rich in *A. cardamomum* has been reported to have protective bioactivity on liver against steatosis, and 2,3,7,8-tetrachlorodibenzo-*p*-dioxin (TCDD) in vivo [30, 31]. Another study revealed relation between antioxidant activity and hepatoprotection [32, 33] which suggests potential effect of *A. cardamomum* on the liver disease related to free radical and other ROS production. The effects of *A. cardamomum* and its fractions on attenuating CCl_4_-induced hepatotoxicity are unknown, with no study of the involvement of antioxidant activity in vitro and in vivo.

Therefore, in this study we evaluated the possible antioxidant properties and hepatoprotective effects of the ethyl acetate fraction obtained from *A. cardamomum* (EAAC) against CCl_4_-induced hepatic injury *in vitro* and in vivo. Furthermore, CYP2E1 gene expression level was investigated to demonstrate the downregulating activity of the EAAC; results were compared to the effects of silymarin, a drug commonly used as a liver therapeutic agent.

## Methods

### Chemicals

2.2-Diphenyl-1-picryl hydrazyl (DPPH), butylated hydroxytoluene (BHT), d(+)-catechin, gallic acid, tannic acid, sodium carbonate, nitrobluetetrazolium (NBT), xanthine, xanthine oxidase, and Folin-Ciocalteu reagent, iron(III) chloride (FeCl_3_), hydrogen peroxide (H_2_O_2_), ascorbic acid were all purchased from Sigma**-**Aldrich (St. Louis, MO, USA) or Merck & Co (Darmstadt, Germany). For *in vitro* studies, bovine serum albumin, Dulbecco’s Modified Eagle Medium (DMEM), fetal bovine serum (FBS), Dulbecco’s phosphate buffered saline (DPBS), penicillin, and streptomycin were purchased from Hyclone (Logan, UT, USA). Oligo primers were purchased from Macrogen (Seoul, Korea). For in vivo studies, silymarin, olive oil, CCl_4_, Oil Red O, hematoxylin and eosin (H&E) stain, triethanolamide, 5,5’-dithiobis(2-nitrobenzoic acid) (DTNB), glutathione (GSH), superoxide dismutase (SOD), 2-thiobarbituric acid (TBA), and sodium azide were purchased from Sigma-Aldrich.

### *A. cardamomum* and extraction

Dried *A. cardamomum* seeds were purchased from Dongwoodang (Yeongcheon-si, Korea). The seeds were ground finely using a mixer grinder and the resulting powder was extracted by a 3-day immersion in 70 % ethanol. The extract was evaporated using a rotary evaporator (Büchi, Flawil, Switzerland) prior to sequential fractionation using hexane, dichloromethane, and ethyl acetate applied with an extraction funnel. Each fraction was concentrated and dried using the aforementioned rotary evaporator. The extract was harvested and used as samples.

### Cell culture and viability assay

HepG2 human liver carcinoma cells were cultured in DMEM supplemented with 10 % FBS, 100 U/ml penicillin and streptomycin. Cells were incubated at 37 °C in a humidified environment containing 5 % CO_2_. Cells were subcultured at 70-80 % confluence and seeded at a density of 1 × 10^5^ cells/well in 96-well plates. After 24 h, the medium changed to FBS-free DMEM. After 24 h pretreatment of EAAC samples (dissolved in DMSO), the medium was changed to DMEM containing 8 mM CCl_4_. The cells were incubated in 37 °C in the humidified CO_2_ incubator for 2 h followed by cell viability determined using the EZ-Cytox cell viability assay kit (Daeil Lab Service, Seoul, Korea) as described by the manufacturer. Briefly, 10 μl of the EZ-Cytox reagent was added to each culture well of a 96-well microplate and incubated at 37 °C in the humidified CO_2_ incubator for 2 h. After incubation, optical density (OD) of the supernatant was measured at a wavelength of 450 nm using a microplate reader.

### Determination of total phenolic, flavonoids and tannins

#### Tannin content

Tannin content was measured using the Folin-Denis method [34]. Fifty microliters of extract was made up to 7.5 ml by the addition of distilled water. Then, 0.5 ml of Folin Denis reagent and 1 ml of Na_2_CO_3_ were added and mixed. The volume was made up to 10 ml using distilled water. The absorption was recorded at 700 nm. Tannic acid and distilled water was used as standard and blank, respectively.

#### Phenol content

Phenol content was measured by the Folin-Ciocalteu method [35]. A sample aliquot 40 μl was added to 200 μl of Folin-Ciocalteu reagent along with 1160 μl of distilled water and mixed. The mixture was incubated for 3 min at room temperature prior to the addition of 600 μl of 2 % sodium carbonate. After 2 h incubation in the dark, the mixture was aliquoted into wells of a 96 well plate and the OD was measured at 765 nm. Gallic acid and distilled water was used as standard and blank, respectively.

#### Flavonoid content

Total flavonoid content for samples was determined by the aluminium chloride colorimetric method [36] with slight modification. 1 ml of water was added to 250 μl samples in a tube. At zero time, 75 μl of 5 % NaNO_2_ was added to the tube. After 5 min, 0.3 ml of 10 % AlCl_3_ was added and incubated for 6 min. After, 0.5 ml of 1 M NaOH was added to the mixture. Absorbance was read at 510 nm with water as the blank. Various concentrations of (+) catechin hydrate solution was used as standard.

#### *In vitro* antioxidant properties

To determine free radical scavenging activities of sample, 40 μl of various concentrations of sample was added to 760 μl solution of 0.3 mM DPPH dissolved in ethanol. An equal amount of ethanol and DPPH served as control. After 30 min incubation in the dark, the absorbance was recorded at 517 nm. The experiment was performed in triplicate and the activity was presented as percentage of scavenged radical. To determine superoxide anion scavenging activities of sample a slight modification of a prior protocol was used [37]. In brief, each sample was mixed with 30 mM EDTA (pH 7.4), 3 mM hypoxanthine in 50 mM sodium peroxide, and 1.42 mM NBT. The mixture was incubated for 3 min at room temperature following the addition of xanthine oxidase and increased volume to to 3 ml with phosphate buffer (pH 7.4). The mixture was incubated for 20 min at room temperature and absorbance at 560 nm was measured using a spectrophotometer. To determine hydroxyl radical scavenging activity, cloned pBR322 plasmid DNA from transformed bacteria was used in assay. Supercoiled (SC) pBR322 plasmid DNA (2.0 μg) was mixed with various concentrations of EAAC. The fenton’s reagent (80 μM FeCl_3_, 0.3 mM H_2_O_2_, 50 μM ascorbic acid) was added to samples and volume brought up to 20 μl. The mixture was incubated at 37 °C for 30 min. Samples were loaded into agarose gel and photographed under UV illuminator.

### Animals and experimental design

Six-week-old specific pathogen-free male Sprague–Dawley rats (*n* = 30) purchased from Koatech (Gyeonggi-do, Korea) received standard normal diet and water ad libitum. The animals were acclimatized to 12 h light/dark cycles for 7 days prior to the experiments. They were divided randomly into five groups of six rats: negative control (olive oil; 1 ml/kg), CCl_4_ (1 ml/kg, dissolved 1:1 in olive oil), CCl_4_ + Silymarin (50 mg/kg, dissolved in olive oil), CCl_4_ + low dose EAAC (100 mg/kg, dissolved in olive oil), and CCl_4_ + high dose EAAC (200 mg/kg, dissolved in olive oil). All treatments were administrated every 72 h for 5 weeks. Bodyweight was recorded weekly. The day after the final treatment, all animals were starved overnight and sacrificed. Whole blood was collected from the abdominal aorta and the liver was harvested under zoletil anesthesia. All protocols for animal experiments were approved by the ethics committee of Dongguk University (No. 2014–09110).

### Serum biochemistry

After sacrifice, the collected blood was immediately centrifuged at 3000 rpm for 20 min. The supernatant was stored at 4 °C until analyses for alanine transaminase (ALT), aspartate transaminase (AST), and alkaline phosphatase (ALP) using commercial kits (Company, Asan, Korea) according to the manufacture’s protocols. As well, OD was determined using a spectrophotometer.

### Determination of lipid peroxidation

Lipid peroxidation level of rat liver was assessed by a previously described malondialdehyde (MDA) assay protocol [38] with slight modification*.* In brief, rats were sacrificed and the liver was isolated after blood perfusion. Liver tissue was homogenized with 1.15 % KCl (9:1, w/w). Aliquots (400 μl) of homogenate were mixed with TBA to a final concentration of 8.1 %. The reactant was heated to 95 °C for 1 h prior to the addition of 1 ml of distilled water and a 5 ml solution of n-butanol and pyridine (15:1). The reactant was centrifuged at 3000 rpm for 30 min. The supernatant was transferred to wells of a 96-well plate and the OD was measured at 532 nm. Various concentrations of 1,1,3,3-tetraethoxy propane were used as standard.

### Intracellular antioxidant enzymes detection

Total Sulfhydryl (SH) level of liver tissue was measured using a prior protocol [39]. In brief, 20 μl of liver sample or standard was mixed with 75 μl of Tris–HCl (pH 8.2) and 25 μl of DTNB (3 mM) in methanol prior to the addition of 400 μl of methanol. Then mixture was spin down at 3000 g for 5 min at room temperature. The sample was transferred to well of a microplate and absorbance was read at 412 nm. Different concentrations of GSH were used as standard. The experiments were conducted in triplicate. SOD activity of liver tissue was investigated using NBT [40]. Briefly, 0.1 mM xanthine, 0.1 mM EDTA, and 25 uM NBT were dissolved in 60 uM sodium bicarbonate buffer. Sample and reaction buffer was mixed in a 1:9 (w/w) ratio. SOD was used as standard. One unit of SOD was equivalent to the amount of enzyme that inhibited the rate of the CYP-catalyzed reaction by 50 %.

### Histology

Liver tissues were frozen using a frozen section compound (Leica, Jena, Germany) and sectioned using a model CM 1860 cryotome (Leica). Slide sections were fixed with 10 % formaldehyde and deparaffinized and stained with H&E or Oil Red O stain. Microscopic images were taken under 200 × concentrations using a DFC 480 microscope system (Leica).

### Quantitative real-time polymerase chain reaction (RT-PCR) and conventional PCR

A portion of each liver tissue was stored in RNA Later solution (Life Technologies, Carlsbad, CA, USA) at −80 °C for investigation of mRNA expression. Liver mRNA was isolated from liver tissues using Trisure (Bioline, Taunton, MA, USA) following the manufacturer’s protocol. Isolated mRNA was checked for RNA integrity and cDNA was synthesized. PCR amplification comprised 10 min at 95 °C, 45 denaturation cycles at 95 °C for 10 s, annealing at 52 °C for 30 s, and extension at 72 °C for 15 s. This was followed by melting curve analysis. Every Ct value and Second Derivative Max quantification was checked. Results were analyzed using Light Cycler software (Roche Applied Science, Basel, Switzerland). Primer sequences used for RT-PCR were as follows: GST sense 5’- GCCTTCTACCCGAAGACACCTT - 3’ and antisense 5’ - GTCAGCCTGTTCCCTACA - 3’, SOD sense 5’ - AGGCCGTGTGCGTGCTGAG - 3’ and antisense 5’ - CACCTTTGCCCAAGTCATCTGC - 3’, CYP2E1 sense 5’ - ATGTCATCCCCAAGGGTACA - 3’ and antisense 5’ - AGGCCTTCTCCAACACACAC - 3’, GAPDH sense 5’ - GGCACAGTCAAGGCTGAGAATG - 3’ and GAPDH antisense 5’ – ATGGTGGTGAAGACGCCAGTA - 3’.

Conventional PCR for CYP2E1 gene was conducted with 30 amplification cycles of PCR consisting of denaturation at 95 °C for 1 min, annealing at 52 °C for 1.5 min, elongation at 72 °C for 2 min. Equal amount of PCR product was then loaded and performed electrophoresis on 1 % agarose gel for 30 min. The relevant expression level was then visualized by UV illuminator (UVP, Cambridge, UK).

### Statistical analyses

The results are expressed as mean ± standard deviation (SD). Experimental data were analyzed using Graph Pad prism version 5.0 software (Graph Pad, La Jolla, CA, USA). Standard curves were constructed using Excel and Powerpoint software (Microsoft, Redmond, WA, USA). All samples were compared with a standard graph using analysis of variance and Student’s *t*-test. A p-value < 0.05 was considered statistically significant.

## Results

### Phytochemical analysis and antioxidant activity

Among the fractions obtained, EAAC displayed the highest content of polyphenol (72.32 ± 1.22 mg GAE/g dried extract) and flavonoid (4.03 ± 0.05 mg CE/g dried extract). Tannins were most abundant in the hexane fraction (86.12 ± 3.09 mg TAE/g dried extract) followed by the ethyl acetate fraction (51.52 ± 3.32 mg TAE/g dried extract) (Table 1).

**Table 1:** Total phenolic, flavonoid and tannin contents and antioxidatn activity of different extract from *Amomum cardamomum* L

Extract	TPC	TFC	TTC	DPPH	NBT reduction IC_50_ (μg/ml)
	(mg GAE/g extract)	(mg CE/g extract)	(mg TAE/g extract)	IC_50_ (μg/ml)	
Aqua	7.59 ± 0.73	-	13.91 ± 2.43	-	-
Ethanol	24.49 ± 0.13	0.42 ± 0.13	40.78 ± 0.94	249.77 ± 8.09	-
Ethyl acetate	72.32 ± 1.22	4.03 ± 0.05	51.52 ± 3.32	148.51 ± 6.51	21.35 ± 4.94
Buthanol	31.83 ± 0.20	0.42 ± 0.20	20.70 ± 6.31	211.05 ± 2.32	-
Dichloromethane	38.64 ± 1.17	1.62 ± 0.05	39.81 ± 1.46	163.31 ± 8.60	46.15 ± 5.55
Hexane	29.95 ± 1.12	3.75 ± 0.28	86.12 ± 3.09	224.33 ± 5.01	-
BHT	-	-	-	34.78 ± 2.92	-
Ascorbic acid	-	-	-	-	22.02 ± 3.25

*TPC* total phenolic content, *TFC* total flavonoid content, *TTC* total tannin content, GAE gallic acid equivalent, *CE* catechin equivalent, *TAE* tannic acid equivalent

**Fig. 1:**
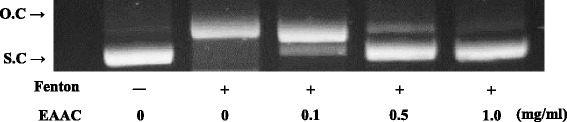
Anti-oxidative activities of ethyl acetate fractions from *Amomum cardamomum* (EAAC) determined by hydroxyl radical assay. The result of DNA nicking assay for hydroxyl radical scavanging activity was illuminated by checking DNA on gel electrophoresis. *O.C* Open cicular DNA nick form, *S.C* Super coil plamid DNA

*A. cardamomum* extracts were fractionated with various solvents. EAAC showed highest efficacy in free radical scavenging activity and superoxide anion scavenging activity (Table 1). EAAC also displayed hydroxyl radical scavenging activity. Fenton’s reagent fragmented plasmid DNA into small size band (Fig. 1). EAAC displayed a dose-dependent protective effect from hydrogen peroxide.

### HepG2 cell proliferation in CCl_4_-induced hepatotoxicity

**Fig. 2:**
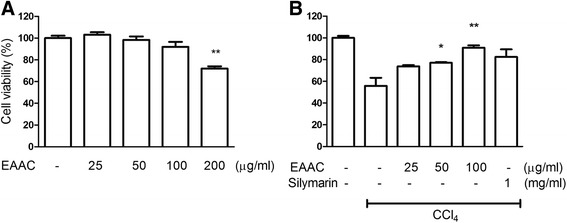
Hepatoprotective effect of EAAC was assessed *in vitro*. **a** HepG2 Cell viability with different concentrations of EAAC was determined after 24 h incubation. **b** Recovered HepG2 cell viability against CCl_4_ by treatment of different concentrations of EAAC and silymarin. The culture media changed with FBS-free DMEM containing CCl_4_ (8 mM) for 2 h. * shows statistically significant differences at *p* < 0.05 from the CCl_4_ group and ** at *p* < 0.01

There was no significant toxicity shown by EAAC treatment to HepG2 cell for 24 h under 100 μg/ml concentration (Fig. 2a). Changing the culture medium to CCl_4_ (8 mM) DMEM for 2 h reduced HepG2 cell viability by 55 % (Fig. 2b). Silymarin pretreatment successfully increased viability of HepG2 cells. Furthermore, viability of cell populations pretreated with EAAC was enhanced upon CCl_4_ treatment in a dose dependent manner, with 90 % preservation of viability using 100 μg/ml EAAC.

### Liver enzyme activities in rat serum levels

**Fig. 3:**
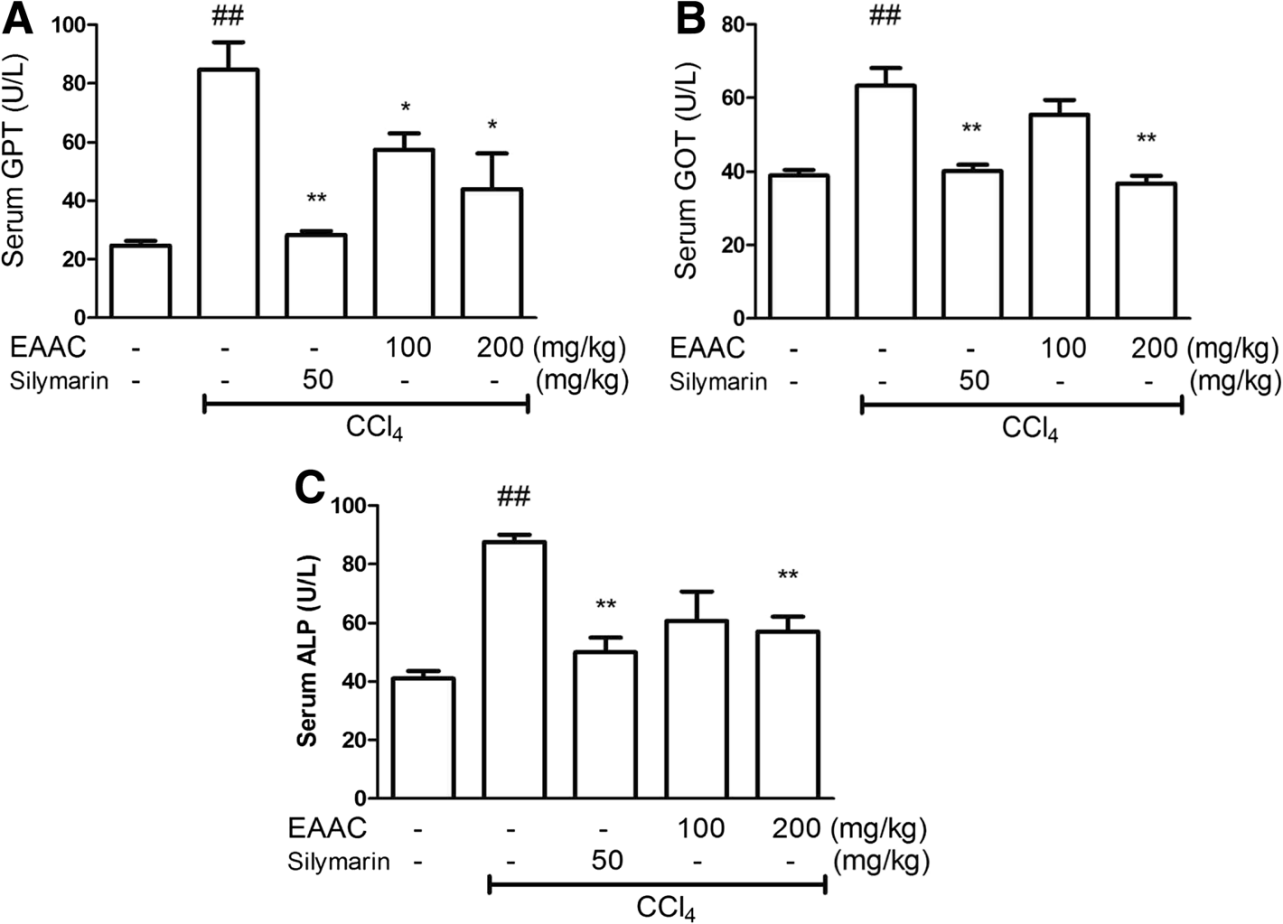
Effect of EAAC on various liver enzyme levels of serum from SD rat in vivo study. **a** GPT enzyme concentration, (**b**) GOT enzyme concentration, (**c**) ALP enzyme concentration were represented as international unit (U/L). ^##^ shows statistically significnat difference at *p* < 0.01 from the untreated group. * shows statistically significant differences at *p* < 0.05 from the CCl_4_ group and ** at *p* < 0.01

All serum enzymes of rats regularly injected with CCl_4_ were significantly increase compared with the normal group. The silymarin group showed significant reductions in serum levels of GPT, GOT, and ALP. Serum GPT activity was also significantly decreased by both concentrations of EAAC, with both results were significant (Fig. 3a; both *p* < 0.05). Serum GOT level was significantly decreased in the EAAC 200 mg/kg group (*p* < 0.01) but not in the 100 mg/kg group (Fig. 3b). Serum ALP levels were markedly decreased by EAAC 200 mg/kg administration (Fig. 3c; *p* < 0.05).

### Histological assessment of liver tissues

**Fig. 4:**
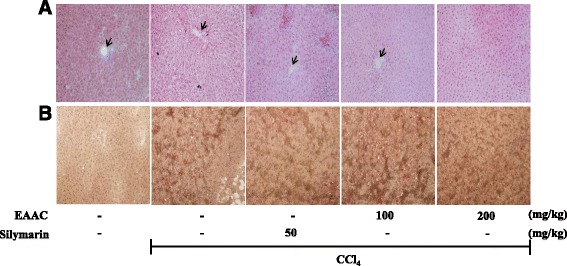
**a**. Hematoxylin-eosin (H&E) stained and (**b**). Oil Red O (ORO) stained sections of liver tissues from SD rat in vivo study. Normal group, CCl_4_ (1 mL/kg) treated group, Silymarin (50 mg/kg) + CCl_4_ treated group, EAAC (100 mg/kg) + CCl4 group and EAAC (200 mg/kg) + CCl_4_ group from left to right. Pictures were taken by microscope under 200× magnification. Pictures show nearby area of central vein (arrow head)

Livers were isolated from rats after sacrifice. H&E staining of liver sections of each group revealed severely damage by CCl_4_ injection in tissue around the hepatic vein (Fig. 4a). Silymarin treatment resulted in relatively less necrosis and liver tissue collapse. Both concentrations of EAAC ameliorated hepatic damage induced by CCl_4_. Oil red O (ORO) staining of liver section is presenting lipid accumulation caused by CCl_4._ The ORO stain appeared in lesser portion of liver section in EAAC 200 mg/kg and silymarin groups compared with CCl_4_ group.

### Reduction of lipid peroxidation

**Fig. 5:**
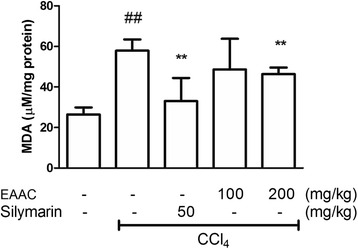
Effect of EAAC treatment on MDA levels in rat liver. Liver tissue from each group was homogenized with 1.15 % KCl buffer (9:1, w/w) and centrifuged. After centrifuged, supernatant was collected to investigate MDA level. ^##^ shows statistically significnat difference at *p* < 0.01 from the untreated group. * shows statistically significant differences at *p* < 0.05 from the CCl_4_ group and ** at *p* < 0.01

To investigate the effect of EAAC administration on lipid peroxidation, a MDA assay was carried out with liver tissue of each group. CCl_4_ injection induced the accumulation of lipid in liver, thus producing peroxidation product (Fig. 5). Silymarin treatment significantly reduced the products of lipid peroxidation. EAAC administration decreased amount of lipid peroxidation contents in liver tissue in a dose-dependent manner. Administration of EAAC 200 mg/kg significantly decreased MDA product (*p* < 0.01), but EAAC 100 mg/kg did not.

### Detoxification of CCl_4_-induced liver damage

**Fig. 6:**
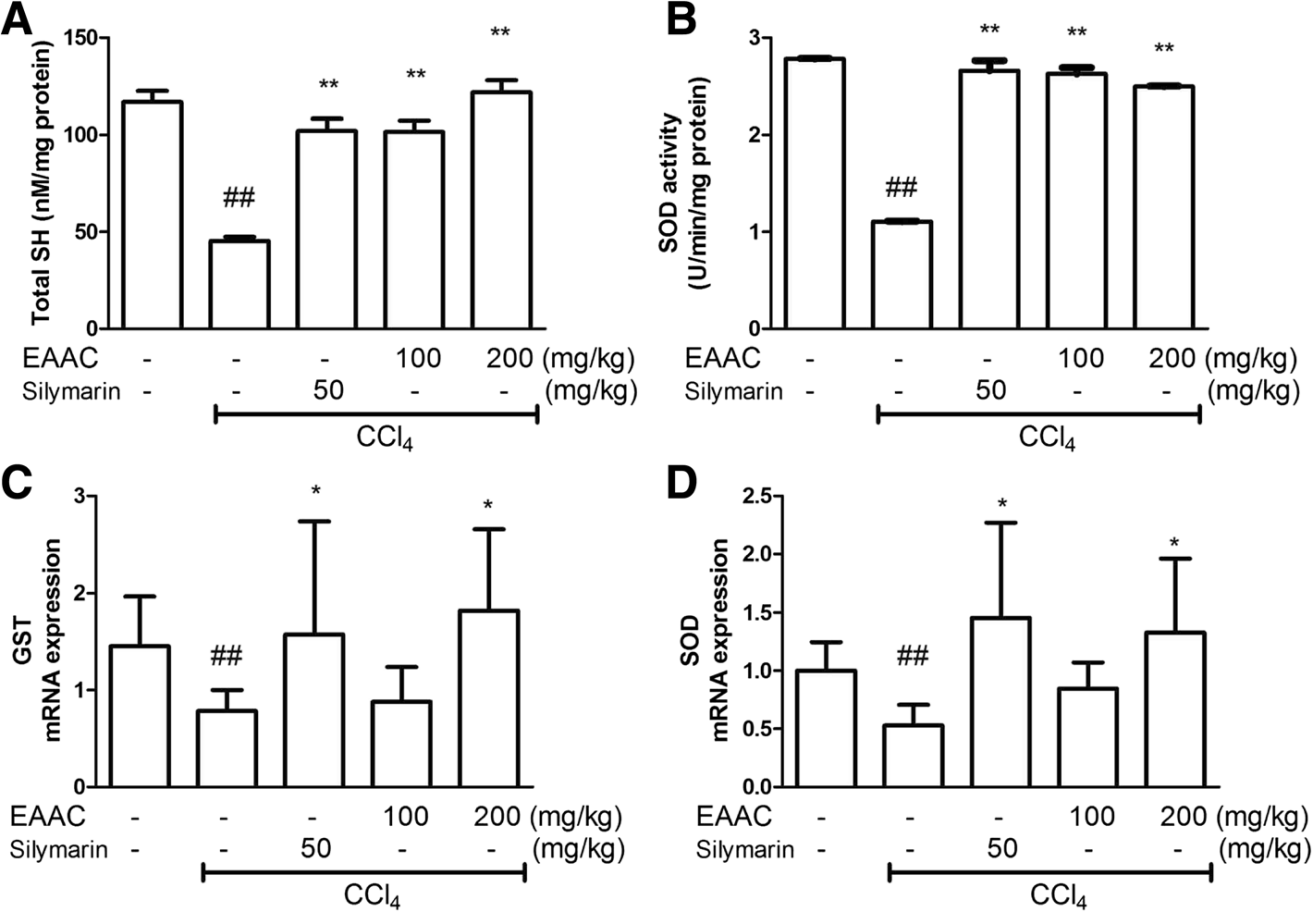
Effect of EAAC treatment on total SH levels, SOD activity and anti-oxidative mRNA gene levels in rat liver. Liver tissue was homogenized and centrifuged. After centrifuged, supernatant was collected to investigate (**a**) total SH level and (**b**) SOD activity. For gene expression analysis, liver tissue was homogenized and lysed with Trisure to isolate mRNA. After cDNA synthesized, real-time PCR was conducted to investigate mRNA expression level. **c** GST expression level and (**d**) SOD expression level. ^##^ shows statistically significnat difference at *p* < 0.01 from the untreated group. * shows statistically significant differences at *p* < 0.05 from the CCl_4_ group and ** at *p* < 0.01

As toxicity induced by CCl_4_ was attenuated by EAAC administration, the concentration of liver detoxification enzyme was investigated. The EAAC effect on total sulfhydryl (SH) and SOD activities was assessed (Figs. 6a and b). CCl_4_ injection significantly reduced total SH and SOD activities. Silymarin treatment successfully recovered both enzyme levels to almost the normal levels (*p* < 0.05). However, there was a notable increase in both total SH and SOD activities by both concentrations of EAAC (*p* < 0.05). Gene expression levels of GST and SOD in the liver were assessed with real-time quantitative PCR. GST and SOD expression was suppressed by CCl_4_ injection, but was significantly escalated in rats treated with silymarin EAAC 200 mg/kg (Fig. 6c, d; *p* < 0.05).

### Reduction of CYP2E1 expression levels

**Fig. 7:**
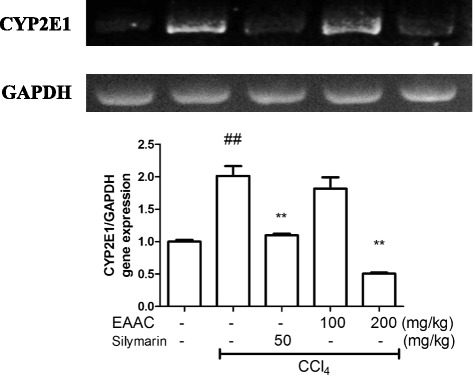
Effect of EAAC treatment on CYP2E1 mRNA levels in rat liver. Liver tissue was homogenized and lysed with Trisure to isolate mRNA. After cDNA synthesized, both conventional PCR and real-time PCR was conducted to investigate mRNA expression level. The result of conventional PCR (CYP2E1 and GAPDH) was illuminated by gel electrophoresis. ^##^ shows statistically significnat difference at *p* < 0.01 from the untreated group. * shows statistically significant differences at *p* < 0.05 from the CCl_4_ group and ** at *p* < 0.01

To investigate the mechanism related with the effect of EAAC treatment, we performed real time quantitative PCR. CCl_4_ injection increased CYP2E1 gene expression by 101 % compared with the normal group. Silymarin treatment reduced this increment significantly (*p* < 0.01). Treatment with EAAC 100 mg/kg produced no significant difference, but whereas the 200 mg/kg concentration significantly reduced CYP2E1 expression (Fig. 7; *p* < 0.01). This was also supported by similar result of conventional PCR product loaded on agarose gel (Fig. 7, upper panel).

## Discussion

The data presented in this study demonstrate that *A. cardamomum* protects against CCl_4_-induced acute liver injury. CCl_4_ is an extensively studied hepatotoxin that is converted CCl_3_ including trichloromethyl (CCl_3_·, CCl_3_OO·) free radicals [15]. Free radical and oxidative stresses have been associated with numerous liver diseases, such as cirrhosis, genotoxicity of hepatic tissue, and hepatic carcinoma [1, 41]. Several endogenous enzymatic and non-enzymatic systems are needed to protect the liver from free radicals [42]. Natural plant-derived antioxidants protect cellular detoxification systems from the harmful responses of excessive oxidation converted free radicals from CCl_4_ [20, 43].

The antioxidant properties of the *A. cardamomum* ethanol extract and organic fractions were tested using various *in vitro* systems. Especially, the ethyl acetate fraction from *A. cardamomum* (EAAC) strongly inhibited formation of DPPH free radicals, superoxide anions, and hydroxyl radicals. The relatively high antioxidant capacity was attributable to the abundance of polyphenol and flavonoid compounds in ethyl acetate fraction from crude extract of natural plants. Similarly, a recent study [44] found that the ethyl acetate fraction of *Crescentia cuhete* leaves and stem bark possess a stronger antioxidant capacity than other fractions in a rapid *in vitro* assay. It is clear that ethyl acetate fractions from natural plants that contain phenolic compounds and flavonoids have superior antioxidant properties. In addition, the treatment of HepG2 hepatocarcinoma cells with CCl_4_ presently resulted in decreased cell viability. This observation agrees with previous reports using various cancer cell lines [9, 45]. On the other hand, pretreatment with EAAC significant recovered cell viability, perhaps due to the reduction of cytotoxicity.

Various recent studies have demonstrated that natural plant-derived phytochemicals protect the liver against CCl_4_-induced damage, such as cirrhosis, steatosis, and hepatic fibrosis [7, 20, 46]. This was presently implied by the significant decreases in serum levels of ALT, AST, and ALP in liver tissue that displayed protection against CCl_4_-induced degeneration. The decrease levels of these serum enzymes correlates with fewer necrotic lesions or histopathological injury, and lipid peroxidation in liver tissue [12, 47]. Moreover, induction of phase II enzymes including GSH, GST, and SOD are important in the balance between oxidative stress and antioxidation in a CCl_4_-induced acute liver injury model [48]. The present results show that exposure to CCl_4_ caused significant increases in serum ALT, AST, and ALP due to hepatic damage in rat liver. However, administration of 100 mg/kg and 200 mg/kg body weight EAAC markedly restored liver physiology, which might be due to the phenolic compounds and total flavonoids. Furthermore, treatment with EAAC increased several phase II enzymes including total SH, GST, and SOD, which were reduced by CCl_4_, likely due to the antioxidant properties of EAAC.

CYP2E1, the most important hepatic cytochrome P450 isoform, is a CCl_4_ converting enzyme that catalyzes production of trichloromethyl free radicals in the liver [15, 49]. The liver is organ that is clearly influenced by CYP2E1, therefore, downregulation of CYP2E1 was expected by decrease of trichloromethyl free radical formation and reduced liver damage, inducing hepatocyte necrosis and hepatocellular injury [50]. In this study, the treatment of rats with CCl_4_ led to a significant overexpression of the CYP2E1 gene compared to control rats. However, treatment with 100 mg/kg and 200 mg/kg body weight EAAC significantly reduced CYP2E1 production. Therefore, it is suggested that *A. cardamomum* has valuable therapeutic potential for liver disease caused by CYP2E1 expression by decreasing CYP2E1 expression in liver.

## Conclusion

The present study clearly demonstrates for the first time the hepatoprotective effect of EAAC against CCl_4_-induced acute liver injury. EAAC treatment significantly alleviated oxidative damage and lipid oxidation through its antioxidant properties. In addition, EAAC downregulated CYP2E1 on hepatotoxic events, comparable to the effect of silymarin in liver tissue. These findings suggest that *A. cardamomum* could be therapeutic as a traditional medicine in acute liver injury models.

## Ethics approval and consent to participate

Present work was approved by the Ethics Committee at the Dongguk University, and all experiments were performed in accordance with the guidelines of the National Animal Care and Use Committee.

## Consent for publication

Not applicable.

## Availability of data and materials

The datasets supporting the conclusions of this article are presented in this main paper.

## Abbreviations

ALP: alkaline phosphatase
BHT: butylated hydroxytoluene
CCl_3_: trichloromethyl radicals
CCl_4_: carbon tetrachloride
CYP450: cytochrome P450
DMEM: Dulbecco’s modified eagle medium
DMSO: dimethyl sulfoxide
DPBS: Dulbecco’s phosphate buffered saline
DPPH: 2.2-Diphenyl-1-picryl hydrazyl
DTNB: 2-nitrobenzoic acid
EAAC: ethyl acetate fraction from *A. cardamomum*
FBS: fetal bovine serum
GOT: glutamic oxaloacetic transaminase
GPT: glutamic pyruvic transaminase
GSH: total glutathione
GST: glutathione S-transferase
H&E: hematoxylin-eosin
MDA: malondialdehyde
NBT: nitrobluetetrazolium
O.D: optical density
ORO: Oil red O
ROS: reactive oxygen species
RT-PCR: real-time polymerase chain reaction
SD: standard deviation
SOD: superoxide dismutase
TBA: 2-thiobarbituric acid
TCDD: 2,3,7,8-tetrachlorodibenzo-*p*-dioxin
